# Suppression of NMDA receptor function in mice prenatally exposed to valproic acid improves social deficits and repetitive behaviors

**DOI:** 10.3389/fnmol.2015.00017

**Published:** 2015-05-27

**Authors:** Jaeseung Kang, Eunjoon Kim

**Affiliations:** ^1^Department of Biological Sciences, Korea Advanced Institute of Science and TechnologyDaejeon, Korea; ^2^Center for Synaptic Brain Dysfunctions, Institute for Basic ScienceDaejeon, Korea

**Keywords:** autism spectrum disorders, valproic acid, NMDA receptors, social interaction, repetitive behavior, memantine

## Abstract

Animals prenatally exposed to valproic acid (VPA), an antiepileptic agent, have been used as a model for autism spectrum disorders (ASDs). Previous studies have identified enhanced NMDA receptor (NMDAR) function in the brain of VPA rats, and demonstrated that pharmacological suppression of NMDAR function normalizes social deficits in these animals. However, whether repetitive behavior, another key feature of ASDs, can be rescued by NMDAR inhibition remains unknown. We report here that memantine, an NMDAR antagonist, administered to VPA mice rescues both social deficits and repetitive behaviors such as self-grooming and jumping. These results suggest that suppression of elevated NMDAR function in VPA animals normalizes repetitive behaviors in addition to social deficits.

## Introduction

Autism spectrum disorders (ASDs) are neurodevelopmental disorders characterized by social defects and repetitive behaviors. Recent genetic studies have identified a large number of genetic variations associated with ASDs (Huguet et al., 2013; Jeste and Geschwind, 2014). In contrast, our understanding of the mechanisms underlying the development of diverse ASD symptoms is limited, although recent studies have begun to suggest candidate mechanisms at the molecular, synaptic and circuit levels, including altered transmission at excitatory and inhibitory synapses and the balance between them (Spooren et al., 2012; Won et al., 2013; Krumm et al., 2014; Gao and Penzes, 2015).

Valproic acid (VPA), an antiepileptic agent, is well known for its teratogenic side effects, including neural tube defects, facial abnormalities, reduced intelligence, and high risk of ASDs (Christianson et al., 1994; Williams and Hersh, 1997; Moore et al., 2000; Williams et al., 2001; Rasalam et al., 2005; Chomiak et al., 2013; Christensen et al., 2013). The causal relationship between prenatal exposure to VPA and the development of ASD symptoms is supported by a large number of animal studies (Ingram et al., 2000; Narita et al., 2002; Miyazaki et al., 2005; Schneider and Przewlocki, 2005; Schneider et al., 2007, 2008; Markram et al., 2008; Snow et al., 2008; Dufour-Rainfray et al., 2010; Gandal et al., 2010; Roullet et al., 2010, 2013; Kim et al., 2011; Roullet and Crawley, 2011; Chomiak et al., 2013).

Several candidate mechanisms have been suggested to explain how VPA enhances the risk of ASDs, including increased acetylation of histone proteins (Fukuchi et al., 2009; Marinova et al., 2009; Foley et al., 2012; Kataoka et al., 2013; Moldrich et al., 2013); excessive proliferation of neural progenitor cells (Go et al., 2011, 2012); altered synaptic development, transmission and plasticity (Rinaldi et al., 2007; Kolozsi et al., 2009; Roullet et al., 2010; Walcott et al., 2011; Sui and Chen, 2012; Banerjee et al., 2013; Bristot Silvestrin et al., 2013; Kim et al., 2013, 2014g; Lin et al., 2013; Kumamaru et al., 2014; Martin and Manzoni, 2014; Nicolini et al., 2015); and disrupted neuronal excitability and neural network activity or formation, such as local hyperconnectivity in the cortex (Markram et al., 2007; Rinaldi et al., 2008a,b; Silva et al., 2009; Chomiak et al., 2010; Chomiak and Hu, 2013).

Although, additional details relating to VPA-induced development of ASDs remain to be investigated, a recent study identified an abnormal increase in NMDA (N-methyl-D-aspartate) receptor (NMDAR) function in the brains of rats prenatally exposed to VPA, as evidenced by upregulation of NMDAR subunits GluN2A and GluN2B, increased NMDAR-mediated synaptic currents, and enhanced postsynaptic long-term potentiation (Rinaldi et al., 2007). In addition, pharmacological suppression of the enhanced NMDAR function in VPA rats improves social deficits (Kim et al., 2014g). These results collectively establish an association of NMDAR hyperfunction with social deficits in VPA rats.

On the basis of previous reports that VPA rats and mice display enhanced repetitive behaviors (Schneider and Przewlocki, 2005; Gandal et al., 2010; Mehta et al., 2011; Kim et al., 2014a), and VPA rats show enhanced NMDAR function (Rinaldi et al., 2007), we hypothesized that suppression of NMDAR function in VPA animals might improve repetitive behaviors. We found that memantine, an NMDAR antagonist, rescued enhanced self-grooming and jumping in VPA mice, linking elevated NMDAR function in VPA mice with repetitive behaviors.

## Methods

### Generation of VPA mice

Pregnant C57BL6/J female mice were administered a single subcutaneous injection of sodium valproate (Sigma) in saline (600 mg/kg), or saline alone (VPA-untreated controls), at embryonic day 13.5 (E13.5). All behavioral tests were performed on mice at 8–16 weeks of age. All mice were bred and maintained according to the KAIST Animal Research Requirements, and all procedures were approved by the Committee of Animal Research at KAIST.

### Drug treatment

Memantine in saline (10 mg/kg), or saline alone (control), was administered to VPA or control (VPA-untreated) mice by intraperitoneal (i.p.) injection 30 min before the three-chamber test or measurements of repetitive behaviors. Injected mice were immediately moved to test positions.

### Three chamber test

The three-chamber test of social interaction and social novelty recognition was performed as described previously (Silverman et al., 2010b) with a slight modification (see below for details) in an apparatus with one center chamber (40 × 20 × 22 cm) and two side chambers (40 × 20 × 22 cm) under a light intensity of ~120 lux. The task was composed of four 10-min sessions. In the first session, a test mouse was habituated to the center chamber. In the second session, a test mouse was allowed to explore all three chambers. Before the third session, a stranger mouse (S1; 129/Sv) was placed in a small plastic cage in the left or right chamber, chosen randomly to avoid side preference. In the third session, the subject mouse was allowed to explore all three chambers and cages. Then, a new stranger mouse (S2) was added to the empty cage, after which the subject mouse was allowed to explore the environment. We added the first session prior to the relatively well-known three following sessions (session 2–4), reasoning that it might increase mouse exploration of side chambers relative to center chamber in sessions 3 and 4. Habituation in the center chamber prior to the whole-apparatus habituation has been reported in original papers (Moy et al., 2004; Nadler et al., 2004), which was to make the center chamber a familiar “home base” of the test mouse (Crawley, 2004). In addition, it has been reported that the time of habituations could be flexible (5–30 min)(Crawley, 2004). Lastly, this and similar four-session three-chamber tests have been reported to work for two independent mouse lines with social deficits (Guo et al., 2009; Chung et al., 2015). Stranger mice (8–16-week-old 129/Sv strain males) were habituated to the plastic cage in the three-chamber apparatus for 30 min 24 h before the test, as described previously (Moy et al., 2004). We used the 129/Sv strain (not C57BL6/J) as a stranger, reasoning that it might increase the social interaction, although the lack of such difference has been reported for certain mouse inbred lines (Nadler et al., 2004). Chamber and sniffing time were measured using Ethovision software (Noldus). The preference index (%) was calculated as (S1 − E)/(S1 + E) × 100 for social interaction, and (S2 − S1)/(S2 + S1) × 100 for social novelty recognition, where E denotes empty cage.

### Self-grooming, jumping, and digging

Self-grooming, jumping, and digging behaviors were measured for 10 min in standard, freshly bedded home cages moved to a novel environment (a soundproof room with a light intensity of ~120 lux) 30 min before the test. Each subject mouse was isolated to a home cage 72 h before measurements. Side cameras were used to record all behaviors. Self-grooming was defined as stroking, scratching, or licking head or body parts with any of the forelimbs. Jumping was defined as simultaneous lifting of all four limbs off the ground, excluding movements associated with scrabbling up the cage wall. Digging was defined as movements in which two fore or hind legs were used coordinately to dig out or displace bedding materials. We also used marble burying as an independent measure of digging (Gyertyan, 1995; Deacon, 2006; Thomas et al., 2009). The marble burying test was performed as described previously (Deacon, 2006), using home cages with flattened bedding (~120 lux). Marbles, stainless steel (2 cm in diameter) or glass (1.5 cm in diameter), were placed in a 3 × 7 arrangement with the inter-marble distance of 4 cm. Each mouse in a home cage was habituated to a soundproof behavior room for 30 min right before the test. At the beginning of the test, a mouse was gently placed in a bedded home cage with marbles, and allowed to explore the environment freely for 30 min. After the test, the number of marbles buried to two-third of their depth was counted.

### Forty eight-hour movements

For measurements of mouse movements in a familiar and completely dark (light-off) environment, VPA mice were moved to and singly isolated in a Laboras cage placed in a soundproof room with a 12-h light on-off cycle 48 h before the test. Mouse movements were monitored for the next 48 h using a vibration-sensitive plate placed underneath the Laboras cage and analyzed using Laboras software.

## Statistics

Statistical details are described in Supplementary Table 1.

## Results

### Memantine rescues social interaction in VPA mice

**Figure 1:**
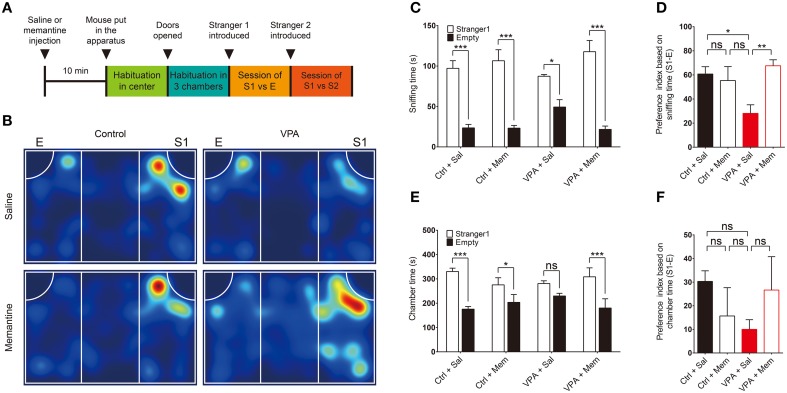
**Memantine rescues social interaction in VPA mice. (A)** Experimental scheme for memantine-dependent rescue of social interaction in VPA mice in the three-chamber test. Mice prenatally (E13.5) exposed to VPA (600 mg/kg) or VPA-untreated control mice (8–16 weeks), were given an i.p. injection of memantine (10 mg/kg) or saline (control) 30 min before encountering a social stranger (S1). E, empty cage; S1, first stranger mouse; S2, second stranger mouse. **(B)** Representative heat map for the movements of saline- or memantine-treated VPA and control (VPA-untreated) mice during the first social interaction session (S1 vs. E). **(C–F)** Quantification of the results in **(B)**, based on the time spent sniffing S1 or E **(C)** and the preference index ([S1 − E]/[S1 + E] × 100) derived from the sniffing parameters **(D)**, or the time spent in the chamber with S1 or E **(E)** and the associated preference index **(F)**. Ctrl, control; VPA, valproic acid; Mem, memantine; Sal, saline. [s. e. m., *n* = 11 for control + Sal/Mem and 10 for VPA + Sal/Mem, ^*^P < 0.05, ^**^P < 0.01, ^***^P < 0.001, ns, not significant; Two-Way ANOVA for **(C)** and **(E)**, and One-Way ANOVA for (**D)** and (**F)**].

VPA mice were generated by subcutaneously injecting pregnant C57B6/J mice on embryonic day 13.5 (E13.5) with a single dose of VPA (600 mg/kg). To determine whether memantine rescues autistic-like phenotypes in VPA mice, we first tested the effect of memantine treatment on social interaction in the three-chamber test (Figure 1A), in which the relative preference of subject mouse for exploration of a stranger mouse trapped in a cage vs. an inanimate object or empty cage was compared (Silverman et al., 2010b).

We found that VPA mice (8–16 weeks) displayed significantly reduced social interaction compared with VPA-untreated mice, as indicated by the relative amount of time spent sniffing a stranger mouse (S1) vs. an empty cage (E), and the preference index derived from these parameters (see figure legend for details) (Figures 1B–D; Supplementary Table 1). When measured using the parameter, time spent in the chamber, as an alternative to sniffing, the reduced social interaction in VPA mice was evident but not as strong as that based on sniffing time (Figures 1E,F), a difference that might be explained by the fact that VPA mice often spent time in locations of the chamber away from the small cage.

Administration of memantine (10 mg/kg) to VPA mice 30 min before the three-chamber test significantly attenuated the reduction in social interaction, restoring this behavior to levels comparable to those in saline-treated control (VPA-untreated) mice (Figures 1B–F). In contrast, memantine treatment of control (VPA-untreated) mice had no effect on social interaction (Figures 1B–F). These results suggest that memantine rescues social interaction in VPA mice, similar to previous findings in VPA rats (Kim et al., 2014g).

**Figure 2:**
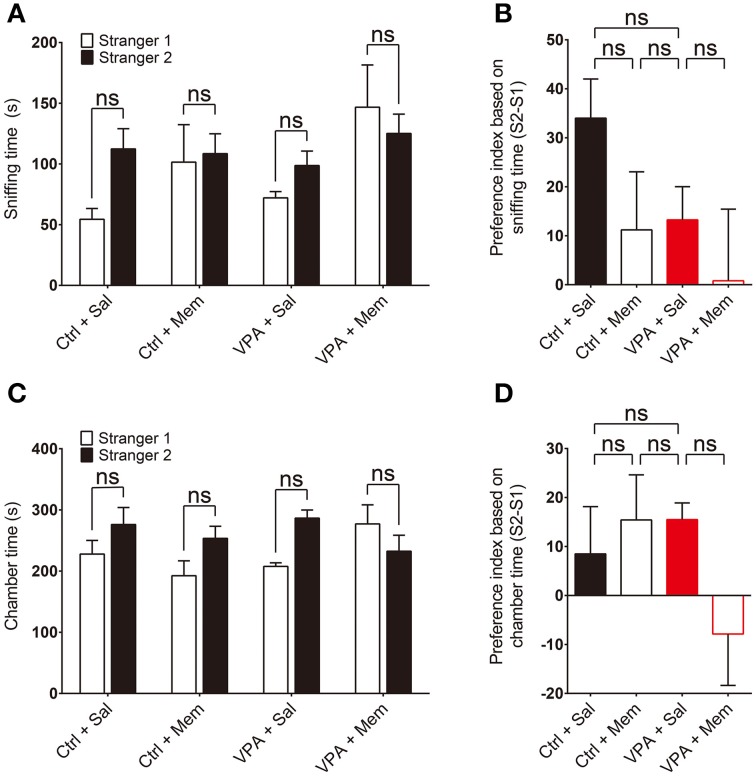
**Memantine has no effect on social novelty recognition in VPA mice. (A–D)** Quantification of the social interactions of saline or memantine-treated VPA and control (VPA-untreated) mice (8–16 weeks) during the second social interaction session (S2 vs. S1), based on the time spent sniffing S2 or S1 **(A)** and the preference index ([S2 − S1]/[S2 + S1] × 100) derived from the sniffing parameters **(B)**, or the time spent in the chamber with the S2 or S1 cage **(C)** and the associated preference index (**D**). [s. e. m., *n* = 11 for control + Sal/Mem and 10 for VPA + Sal/Mem, ns, not significant; Two-Way ANOVA for **(A)** and **(C)**, and One-Way ANOVA for **(B)** and **(D)**].

We next tested social novelty recognition in VPA mice in a subsequent three-chamber test session by adding a new stranger mouse (S2) to the empty cage and allowing the subject mouse to explore S2 or S1 (old stranger). VPA mice showed a tendency for reduced social novelty recognition compared with VPA-untreated control mice, quantified by sniffing time and preference index, although this difference did not reach statistical significance (Figures 2A,B; Supplementary Table 1). In addition, memantine treatment did not significantly change social novelty recognition (Figures 2A,B). Similar results were obtained using chamber time as the measured parameter (Figures 2C,D). These results suggest that prenatal VPA exposure does not alter social novelty recognition in mice and memantine treatment has no effect on it. This is unlike results obtained with VPA rats, in which VPA induces memantine-sensitive impairment of social novelty recognition (Kim et al., 2014g), a difference that likely reflects a difference between species.

### Memantine rescues repetitive self-grooming and jumping in VPA mice

**Figure 3:**
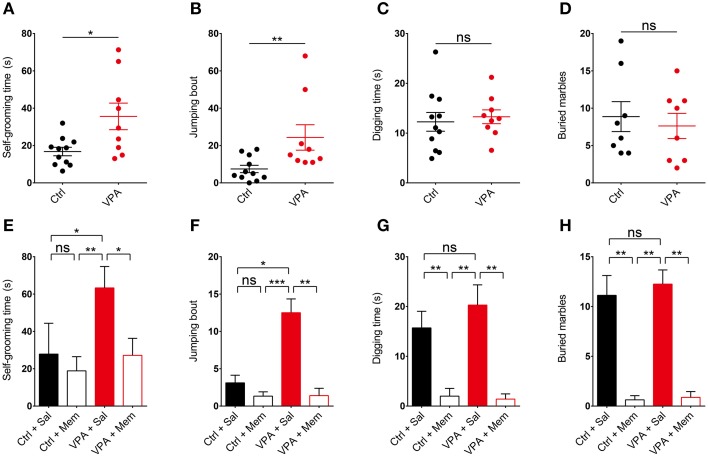
**Memantine rescues repetitive self-grooming and jumping in VPA mice. (A–D)** VPA mice (8–16 weeks) display enhanced self-grooming and jumping, but normal digging in a novel and bright (~120 lux) environment, as measured by increased levels of self-grooming time, jumping bouts, digging time, and number of marbles buried (stainless steel, 2 cm in diameter) in VPA mice moved to a novel soundproof room 30 min before the test. (s. e. m., *n* = 11 for control (VPA-untreated) mice and 9 for VPA mice, ^*^P < 0.05, ^**^P < 0.01, ns, not significant; unpaired Student's *t*-test for self-grooming and digging, Mann–Whitney test for jumping). **(E–H)** Memantine (10 mg/kg, i.p.) administered to VPA mice 30 min before the test rescues repetitive self-grooming and jumping in VPA mice, but has no effect on control (VPA-untreated) mice. Note that memantine strongly suppresses digging in both VPA and control (VPA-untreated) mice. (s. e. m., *n* = 9 for control + Sal/Mem and 10 for VPA + Sal/Mem, ^*^P < 0.05, ^**^P < 0.01, ^***^P < 0.001, ns, not significant; Kruskal–Wallis test, Wilcoxon test, and Student's *t*-test).

We next tested if memantine rescues repetitive behavior in VPA mice. We first tested whether VPA mice display repetitive behaviors by moving them in their home cages to a novel soundproof room with a bright (~120 lux) light 30 min before the test. We found that VPA mice (8–16 weeks) displayed increased repetitive self-grooming and jumping in the novel environment compared with control (VPA-untreated) mice (Figures 3A,B; Supplementary Table 1). This result is similar to the enhanced self-grooming observed in VPA mice in the same genetic background (C57BL/6) (Gandal et al., 2010; Mehta et al., 2011), but contrasts with the unaltered self-grooming in VPA mice in a different genetic background (ICR) (Kim et al., 2014a).

In the next set of experiments, we treated VPA mice with memantine (10 mg/kg, i.p.) 30 min before the test. We found that memantine rescued self-grooming and jumping in these mice, restoring these behaviors to levels comparable to those in saline-treated control (VPA-untreated) mice (Figures 3E,F). Memantine had no effect on self-grooming or jumping in control (VPA-untreated) mice. These results suggest that memantine rescues self-grooming and jumping in VPA mice.

### VPA mice do not display repetitive digging, and memantine strongly suppresses digging behavior

VPA mice on both C57BL/6 and ICR backgrounds have been shown to display increased digging, as quantified by the number of marbles buried (Mehta et al., 2011; Kim et al., 2014a), or manual counts of digging episodes (Kim et al., 2014a). In our study, however, VPA mice did not show enhanced digging, as measured by manual counting of digging or the number of marbles buried (Figures 3C,D; Supplementary Figure 1). Two distinct types of marbles with different size and texture, stainless steel (2 cm in diameter) and glass marbles (1.5 cm in diameter), gave similar results.

Notably, treatment with memantine (10 mg/kg, i.p.) 30 min before the test strongly suppressed digging behavior in both VPA and control (VPA-untreated) mice (Figures 3G,H), similar to the previously reported memantine (10 mg/kg, i.p.; 30 min prior to the test)-induced strong suppression of marble burying in ICR mice (Egashira et al., 2008). These results indicate that VPA mice do not show increased repetitive digging under our experimental conditions, and that memantine strongly suppresses digging behavior regardless of VPA-treatment status.

### VPA mice do not display repetitive self-grooming in a familiar and dark environment

Repetitive self-grooming and jumping in VPA mice described above was measured in a novel and bright environment, which might cause novelty-induced grooming (Spruijt et al., 1992; van Erp et al., 1994). In order to test whether a familiar and dark (light-off) environment could induce repetitive behavior in VPA mice, we moved mice to recording cages in a soundproof room and habituated them to the novel environment for 48 h. The mice were maintained under a 12-h light on/off cycle, and their self-grooming and other movements were recorded for 48 h using a vibration-sensitive sensor placed beneath the cage.

**Figure 4:**
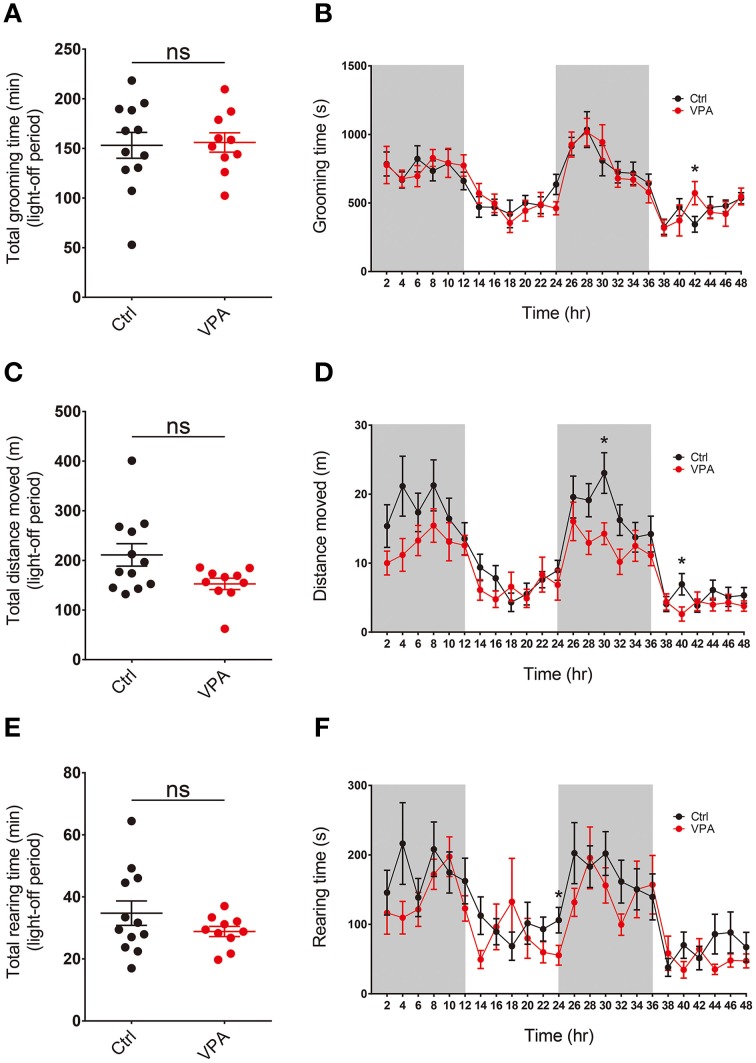
**VPA mice do not display repetitive self-grooming or altered locomotor or rearing activity in a familiar and dark environment. (A–F)** VPA mice (8–16 weeks) do not display significant alterations in self-grooming, locomotor activity or rearing movement, as measured by continuous 48-h monitoring of mouse movements in Laboras cages. Values in (**A)**, (**C)**, and (**E**) are averages of results from two light-off periods. (s. e. m. *n* = 12 for control and 10 for VPA, ^*^P < 0.05; Student's *t*-tests).

We found that the overall level of self-grooming in VPA mice during light-off periods was similar to that observed in control (VPA-untreated) mice (Figures 4A,B; Supplementary Table 1). In addition, levels of locomotor activity and rearing movements were similar between VPA and control (VPA-untreated) mice (Figures 4C–F). These results suggest that VPA mice do not display enhanced repetitive self-grooming or other types of abnormal behaviors in a familiar and dark environment, results dissimilar to those obtained in a novel and bright environment.

## Discussion

In the present study, we demonstrated that suppression of NMDAR function by memantine improves social deficits and repetitive behaviors in VPA mice. In addition, VPA mice displayed repetitive behavior in a novel and bright environment but not in a familiar and dark environment.

The rescue of impaired social interaction in VPA mice by memantine (Figure 1) is similar to previous reports showing that NMDAR suppression by MK801 (NMDAR antagonist), memantine, or MPEP (an mGluR5 antagonist) rescues social-interaction deficits in VPA rats (Kim et al., 2014g). Therefore, memantine appears to restore social deficits in both VPA mice and rats, likely through suppression of enhanced NMDAR function. These results, together with a recent report that memantine and MPEP rescue social deficits in *IRSp53*^−/−^ mice (Chung et al., 2015), which display enhanced NMDAR function (Kim et al., 2009), further links NMDAR hyperfunction with social deficits.

VPA mice did not display impaired social novelty recognition, although there was a tendency toward a decrease (Figure 2). This contrasts with a recent report that social novelty recognition in the three-chamber test was strongly reduced in VPA rats (Kim et al., 2014g). In addition, memantine treatment had no effect on social novelty recognition in VPA or control mice (Figure 2). This again contrasts with the significant enhancement of social novelty recognition by memantine in both VPA and control (VPA-untreated) rats (Kim et al., 2014g). These differences may be attributable to species-specific differences between rats and mice.

VPA mice showed enhanced repetitive self-grooming and jumping (Figures 3A,B), findings similar to the previously reported increase in repetitive self-grooming in VPA mice (C57BL/6 and ICR strains) (Gandal et al., 2010; Mehta et al., 2011; Kim et al., 2014a) and repetitive/stereotypic-like behaviors in VPA rats (Schneider and Przewlocki, 2005). Notably, these repetitive behaviors were rescued by memantine in VPA mice (Figures 3E,F), suggesting that suppression of the enhanced NMDAR function in VPA mice reverses repetitive behaviors. To the best of our knowledge, this is the first report demonstrating rescue of repetitive behavior by direct NMDAR inhibition in an animal model of ASDs. Our results are in line with a previous report that enhanced self-grooming and marble burying in VPA mice are rescued by MPEP (Mehta et al., 2011), which likely suppresses NMDAR function or downstream signaling indirectly through inhibition of mGluR5, a glutamate receptor subtype known to act synergistically with NMDARs (Jia et al., 1998; Alagarsamy et al., 1999). Another finding relevant to the current results is the rescue of enhanced marble burying in VPA mice (ICR strain) by donepezil (Kim et al., 2014a), a medication known to suppress NMDAR function through multiple mechanisms, including inhibition of acetylcholine esterase, stimulation of α7 nicotinic acetylcholine receptors, and endocytosis of NMDARs (Moriguchi et al., 2005; Shen et al., 2010).

VPA mice in a familiar and completely dark environment showed no alterations in self-grooming, horizontal locomotion or rearing movement (Figure 4), results that contrast with the enhanced self-grooming and jumping in VPA mice placed in a novel and bright environment (jumping in the dark environment could not be measured owing to lack of supporting software). The enhanced self-grooming in the novel and bright environment in VPA mice may represent novelty-induced grooming, as previously reported in rats and mice (Spruijt et al., 1992; van Erp et al., 1994). In addition, the normal levels of self-grooming in a familiar, dark environment may reflect decreased novelty and levels of related stressors (Spruijt et al., 1992; van Erp et al., 1994; Komorowska and Pellis, 2004).

Previous studies have shown that NMDAR function is enhanced in the medial prefrontal cortex (mPFC) region of VPA rats at early stages (P12–16), but not at P30 or P40–50 (Rinaldi et al., 2007; Walcott et al., 2011), and is even reduced at later stages (P110–130) (Martin and Manzoni, 2014). How might these results be reconciled with our hypothesis that suppression of the enhanced NMDAR function in VPA mice rescues social deficits and repetitive behaviors? One possibility is that some brain regions other than the mPFC that are associated with autistic-like phenotypes may have enhanced NMDAR function in VPA mice at stages in which behavioral experiments were performed (P56–112). Indeed, *IRSp53*^−/−^ mice, whose social deficits are normalized by memantine, display enhanced NMDAR function in the hippocampus, but normal NMDAR function in the mPFC (Chung et al., 2015). Similarly, *Shank2*^−/−^ mice lacking exons 6 + 7, whose social deficits are rescued by the NMDAR agonist D-cycloserine, display reduced NMDAR function in the hippocampus, but normal NMDAR function in the mPFC (Won et al., 2012). In addition, *Neuroligin-3*^*R*451*C*^ knock-in mice, which express an ASD-related mutation found in humans and display autistic-like behavioral phenotypes, show enhanced NMDAR function in the hippocampus, but enhanced inhibitory synaptic transmission in the somatosensory cortex (Tabuchi et al., 2007; Etherton et al., 2011). However, given the well-known association of the mPFC with social functions, other brain regions with enhanced NMDAR function in VPA mice, if such regions exist, may be functionally connected to the mPFC. For example, memantine, which suppresses elevated NMDAR function in the *IRSp53*^−/−^ hippocampus, has been shown to suppress neuronal firing in the mPFC, where no NMDAR hyperfunction was observed (Chung et al., 2015).

Lastly, while we have thus far associated excessive NMDAR function with autistic-like phenotypes in VPA mice, the opposite change—limited NMDAR function—has also been associated with autistic-like phenotypes in other animal models. For instance, autistic-like social deficits and repetitive behaviors observed in rats and mice are rescued by pharmacological reagents that elevate NMDAR function (Chubykin et al., 2007; Blundell et al., 2010; Silverman et al., 2010a, 2012; Deutsch et al., 2012; Won et al., 2012; Yadav et al., 2012; Benson et al., 2013; Budreck et al., 2013; Burgdorf et al., 2013; Burket et al., 2013; Huang et al., 2014). Therefore, deviation of NMDAR function in either direction appears to be associated with autistic-like phenotypes, and correcting these deviations may have therapeutic potential (Lee et al., 2015).

In summary, our results indicate that suppression of NMDAR function in VPA mice rescues repetitive behavior as well as social deficits.

### Conflict of interest statement

The authors declare that the research was conducted in the absence of any commercial or financial relationships that could be construed as a potential conflict of interest.

## Supplementary material

Supplementary Figure 1 **VPA mice do not show repetitive digging behavior, as determined by the number of marbles buried**. Glass marbles (1.5 cm in diameter) were used to measure digging. (s. e. m., *n* = 8 for control (VPA-untreated) mice and VPA mice, ns, not significant; Student's *t*-test).

Supplementary Table 1 **Statistical details**.
