# Supplementary figures and images for: Suppression of NMDA receptor function in mice prenatally exposed to valproic acid improves social deficits and repetitive behaviors
Source: Front Mol Neurosci. 2015 May 27;8:17. doi: 10.3389/fnmol.2015.00017 (PMC4444740; doi:10.3389/fnmol.2015.00017)

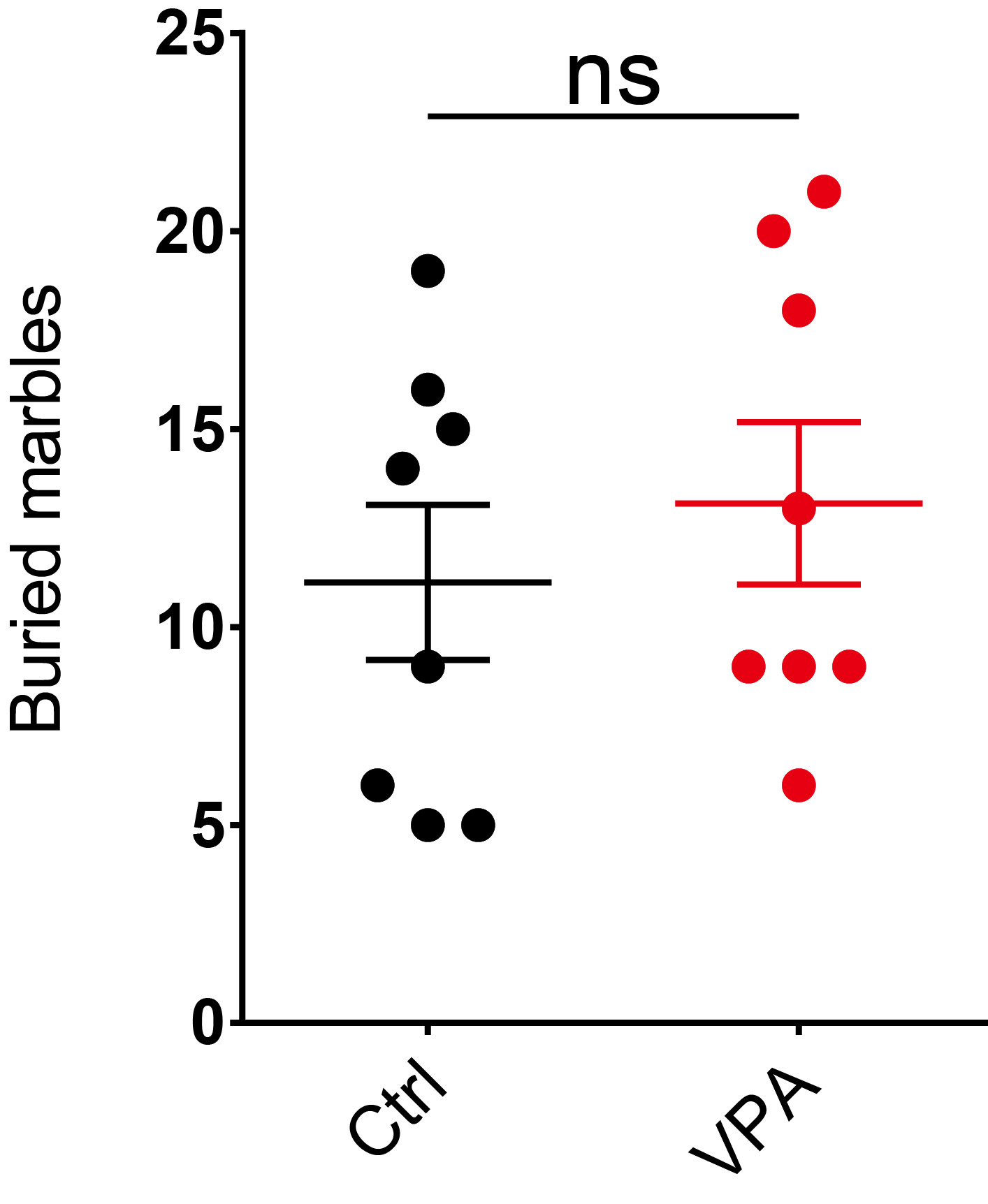

Supplement: Supplementary Figure 1 — VPA mice do not show repetitive digging behavior, as determined by the number of marbles buried. Glass marbles (1.5 cm in diameter) were used to measure digging. (s. e. m., n = 8 for control (VPA-untreated) mice and VPA mice, ns, not significant; Student's t-test). [file Image1.JPEG]
