# Supplementary material for: Suppression of NMDA receptor function in mice prenatally exposed to valproic acid improves social deficits and repetitive behaviors
Source: Front Mol Neurosci. 2015 May 27;8:17. doi: 10.3389/fnmol.2015.00017 (PMC4444740; doi:10.3389/fnmol.2015.00017)
Supplement: Supplementary Table 1 — Statistical details. [file Table1.DOCX]

**Supplementary Table 1. Statistical details.**

| **Figure** | **Panel** | **Assay** | **N number**  **& Age** | **Parameter** | **Mean** $\boldsymbol{\pm}$ **SEM** | **Statistical Test and Significance** | **Conclusion** |
| --- | --- | --- | --- | --- | --- | --- | --- |
| 1 | C | 3-chamber social interaction test  (Stranger 1 vs Empty) | n = 11 for Ctrl  n = 10 for VPA  2-4 month | Sniffing time (s) | Ctrl + Sal S1: 97.16 $\pm$ 9.48  Ctrl + Sal E: 23.53 $\pm$ 4.28  Ctrl + Mem S1: 106.50 $\pm$ 13.69  Ctrl + Mem S2: 23.23 $\pm$ 3.38  VPA + Sal S1: 87.40 $\pm$ 2.09  VPA + Sal E: 49.30 $\pm$ 9.15  VPA + Mem S1: 117.73 $\pm$ 13.84  VPA + Mem E: 21.67 $\pm$ 4.12 | Ordinary two-way ANOVA  (S1 vs E)  Ctrl + Sal: p < 0.0001  Ctrl + Mem: p < 0.0001  VPA + Sal: p = 0.0137  VPA + Mem: p < 0.0001 | Sociability:  Ctrl + Sal = Ctrl + Mem = VPA + Sal > VPA + Mem |
|  | D |  |  | Preference index | Ctrl + Sal: 60.73 $\pm$ 6.087  Ctrl + Mem: 55.30 $\pm$ 11.59  VPA + Sal: 28.07 $\pm$ 7.132  VPA + Mem: 67.58 $\pm$ 4.976 | Ordinary one-way ANOVA  Ctrl + Sal vs VPA + Sal: p = 0.0318  Ctrl + Sal vs Ctrl + Mem: p = 0.9608  Ctrl + Mem vs VPA + Sal: p = 0.0945  VPA + Sal vs VPA + Mem: p = 0.0083 |  |
|  | E |  |  | Chamber time (s) | Ctrl + Sal S1: 330.15 $\pm$ 14.05  Ctrl + Sal E: 175.54 $\pm$ 10.84  Ctrl + Mem S1: 275.27 $\pm$ 29.39  Ctrl + Mem E: 203.58 $\pm$ 32.26  VPA + Sal S1: 280.74 $\pm$ 11.34  VPA + Sal E: 229.68 $\pm$ 11.12  VPA + Mem S1: 308.47 $\pm$ 36.95  VPA + Mem E: 180.10 $\pm$ 37.96 | Ordinary two-way ANOVA  (S1 vs E)  Ctrl + Sal: p < 0.0001  Ctrl + Mem: p = 0.0358  VPA + Sal: p = 0.2009  VPA + Mem: p = 0.0002 |  |
|  | F |  |  | Preference index | Ctrl + Sal: 30.32 $\pm$ 4.47  Ctrl + Mem: 15.67 $\pm$ 11.99  VPA + Sal: 10.01 $\pm$ 4.08  VPA + Mem: 26.66 $\pm$ 14.13 | Kruskal-Wallis test  Ctrl + Sal vs VPA + Sal: p = 0.1305  Ctrl + Sal vs Ctrl + Mem: p > 0.9999  Ctrl + Mem vs VPA + Sal: p = 0.3754  VPA + Sal vs VPA + Mem: p = 0.1395 |  |
| 2 | A | 3-chamber social interaction test  (Stranger 1 vs Stranger 2) | n = 11 for Ctrl  n = 10 for VPA  2-4 month | Sniffing time (s) | Ctrl + Sal S1: 54.46 $\pm$ 8.90  Ctrl + Sal S2: 112.39 $\pm$ 16.61  Ctrl + Mem S1: 101.50 $\pm$ 30.84  Ctrl + Mem S2: 108.52 $\pm$ 16.37  VPA + Sal S1: 72.08 $\pm$ 5.18  VPA + Sal S2: 98.70 $\pm$ 11.91  VPA + Mem S1: 146.74 $\pm$ 34.84  VPA + Mem S2: 125.16 $\pm$ 15.90 | Ordinary two-way ANOVA  (S1 vs S2)  Ctrl + Sal: p = 0.1496  Ctrl + Mem: p = 0.9984  VPA + Sal: p = 0.8345  VPA + Mem: p = 0.9148 | Social Novelty:  Ctrl = VPA  Sal = Mem |
|  | B |  |  | Preference index | Ctrl + Sal: 34.01 $\pm$ 7.986  Ctrl + Mem: 11.19 $\pm$ 11.87  VPA + Sal: 13.23 $\pm$ 6.790  VPA + Mem: 0.796 $\pm$ 14.63 | Ordinary one-way ANOVA  Ctrl + Sal vs VPA + Sal: p = 0.5267  Ctrl + Sal vs Ctrl + Mem: p = 0.4245  Ctrl + Mem vs VPA + Sal: p = 0.9991  VPA + Sal vs VPA + Mem: p = 0.8539 |  |
|  | C |  |  | Chamber time (s) | Ctrl + Sal S1: 227.90 $\pm$ 22.26  Ctrl + Sal S2: 276.12 $\pm$ 27.83  Ctrl + Mem S1: 192.41 $\pm$ 24.39  Ctrl + Mem S2: 253.62 $\pm$ 19.68  VPA + Sal S1: 207.67 $\pm$ 5.92  VPA + Sal S2: 286.54 $\pm$ 13.30  VPA + Mem S1: 277.15 $\pm$ 31.24  VPA + Mem S2: 232.52 $\pm$ 26.09 | Ordinary two-way ANOVA  (S1 vs S2)  Ctrl + Sal: p = 0.4282  Ctrl + Mem: p = 0.2060  VPA + Sal: p = 0.0762  VPA + Mem: p = 0.5508 |  |
|  | D |  |  | Preference index | Ctrl + Sal: 8.49 $\pm$ 9.647  Ctrl + Mem: 15.41 $\pm$ 9.211  VPA + Sal: 15.49 $\pm$ 3.411  VPA + Mem: -7.863 $\pm$ 10.50 | Ordinary one-way ANOVA  Ctrl + Sal vs VPA + Sal: p = 0.9418  Ctrl + Sal vs Ctrl + Mem: p = 0.9396  Ctrl + Mem vs VPA + Sal: p > 0.9999  VPA + Sal vs VPA + Mem: p = 0.2702 |  |
| 3 | A | Homecage activity | n = 11 for Ctrl  n = 9 for VPA  2-4 month | Grooming time (s) | Ctrl: 16.79 $\pm$ 2.278  VPA: 35.61 $\pm$ 7.110 | Unpaired t-test  Ctrl vs VPA: p = 0.0135 | Grooming:  Ctrl < VPA |
|  | B |  |  | Jumping bout | Ctrl: 7.455 $\pm$ 1.960  VPA: 24.33 $\pm$ 6.811 | Mann-Whitney test  Ctrl vs VPA: p = 0.0069 | Jumping:  Ctrl < VPA |
|  | C |  |  | Digging time (s) | Ctrl: 12.27 $\pm$ 1.882  VPA: 13.28 $\pm$ 1.387 | Unpaired t-test  Ctrl vs VPA: p = 0.6815 | Digging:  Ctrl = VPA |
|  | D |  | n = 8 for Ctrl  n = 8 for VPA  2 month | Buried marbles | Ctrl: 8.875 $\pm$ 5.668  VPA: 7.625 $\pm$ 4.779 | Unpaired t-test  Ctrl vs VPA: p = 0.6410 | Digging:  Ctrl = VPA |
|  | E |  | n = 9 for Ctrl  n = 10 for VPA  2-4 month | Grooming time (s) | Ctrl + Sal: 27.84 $\pm$ 16.51  Ctrl + Mem: 18.88 $\pm$ 7.590  VPA + Sal: 63.31 $\pm$ 11.46  VPA + Mem: 27.17 $\pm$ 9.122 | Kruskal-Wallis test  Ctrl + Sal vs VPA + Sal: p = 0.0111  Ctrl + Mem vs VPA + Sal: p = 0.0045  Wilcoxon test  Ctrl + Sal vs Ctrl + Mem: p > 0.9999  Paired t-test  VPA + Sal vs VPA + Mem: p = 0.0222 | Grooming:  Ctrl + Sal = Ctrl + Mem = VPA + Mem < VPA + Sal |
|  | F |  |  | Jumping bout | Ctrl + Sal: 3.111 $\pm$ 1.020  Ctrl + Mem: 1.333 $\pm$ 0.577  VPA + Sal: 12.50 $\pm$ 1.851  VPA + Mem: 1.400 $\pm$ 0.980 | Kruskal-Wallis test  Ctrl + Sal vs VPA + Sal: p = 0.0108  Ctrl + Mem vs VPA + Sal: p = 0.0004  Paired t-test  Ctrl + Sal vs Ctrl + Mem: p = 0.1575  Wilcoxon test  VPA + Sal vs VPA + Mem: p = 0.0020 | Jumping:  Ctrl + Sal = Ctrl + Mem = VPA + Mem < VPA + Sal |
|  | G |  |  | Digging time (s) | Ctrl + Sal: 15.71 $\pm$ 3.335  Ctrl + Mem: 1.992 $\pm$ 1.562  VPA + Sal: 20.29 $\pm$ 4.067  VPA + Mem: 1.407 $\pm$ 1.043 | Kruskal-Wallis test  Ctrl + Sal vs VPA + Sal: p > 0.9999  Ctrl + Mem vs VPA + Sal: p = 0.0008  Wilcoxon test  Ctrl + Sal vs Ctrl + Mem: p = 0.0078  VPA + Sal vs VPA + Mem: p = 0.0020 | Digging:  Sal > Mem |
|  | H |  | n = 8 for Ctrl  n = 8 for VPA  2 month | Buried marbles | Ctrl + Sal: 11.13 $\pm$ 1.986  Ctrl + Mem: 0.625 $\pm$ 0.420  VPA + Sal: 12.25 $\pm$ 1.424  VPA + Mem: 0.875 $\pm$ 0.581 | Kruskal-Wallis test  Ctrl + Sal vs VPA + Sal: p > 0.9999  Ctrl + Mem vs VPA + Sal: p = 0.0012  Ctrl + Sal vs Ctrl + Mem: p = 0.0044  VPA + Sal vs VPA + Mem: p = 0.0018 | Digging:  Sal > Mem |
| 4 | A | Laboras^TM^  (metris) | n = 12 for Ctrl  n = 10 for VPA  2-4 month | Total grooming time (min) (light-off period) | Ctrl: 153.1 $\pm$ 13.07  VPA: 156.0 $\pm$ 9.745 | Unpaired t-test  Ctrl vs VPA: p = 0.8648 | Light-off period long-term grooming:  Ctrl = VPA |
|  | B |  |  | Grooming time (s) | Ctrl  2: 784.63 $\pm$ 87.74  4: 688.02 $\pm$ 59.36  6: 821.76 $\pm$ 95.89  8: 734.65 $\pm$ 72.98  10: 788.79 $\pm$ 102.20  12: 660.99 $\pm$ 64.58  14: 471.13 $\pm$ 74.97  16: 468.80 $\pm$ 58.60  18: 420.07 $\pm$ 100.53  20: 500.94 $\pm$ 54.24  22: 483.33 $\pm$ 61.72  24: 635.09 $\pm$ 74.01  26: 913.80 $\pm$ 65.66  28: 1035.23 $\pm$ 130.77  30: 808.65 $\pm$ 110.91  32: 724.07 $\pm$ 78.14  34: 716.12 $\pm$ 81.90  36: 643.82 $\pm$ 67.33  38: 326.32 $\pm$ 55.32  40: 468.83 $\pm$ 62.19  42: 344.60 $\pm$ 57.08  44: 467.44 $\pm$ 78.27  46: 479.21 $\pm$ 41.82  48: 534.11 $\pm$ 40.79  VPA  2: 776.83 $\pm$ 136.36  4: 676.79 $\pm$ 62.85  6: 695.65 $\pm$ 76.59  8: 828.58 $\pm$ 62.06  10: 792.36 $\pm$ 106.26  12: 773.02 $\pm$ 78.46  14: 570.36 $\pm$ 71.57  16: 497.66 $\pm$ 67.72  18: 355.00 $\pm$ 70.96  20: 443.60 $\pm$ 75.35  22: 489.62 $\pm$ 87.73  24: 461.31 $\pm$ 47.85  26: 926.26 $\pm$ 91.26  28: 1017.21 $\pm$ 100.47  30: 944.73 $\pm$ 126.50  32: 679.16 $\pm$ 64.67  34: 669.43 $\pm$ 45.41  36: 578.92 $\pm$ 78.35  38: 318.64 $\pm$ 58.99  40: 372.23 $\pm$ 113.55  42: 571.74 $\pm$ 84.51  44: 431.89 $\pm$ 46.85  46: 420.08 $\pm$ 89.62  48: 547.87 $\pm$ 60.79 | Multiple t-tests  Ctrl vs VPA  2: p = 0.9609  4: p = 0.9205  6: p = 0.3302  8: p = 0.3497  10: p = 0.9810  12: p = 0.2787  14: p = 0.3560  16: p = 0.7494  18: p = 0.6168  20: p = 0.5348  22: p = 0.9527  24: p = 0.0743  26: p = 0.9109  28: p = 0.9168  30: p = 0.4264  32: p = 0.6708  34: p = 0.6426  36: p = 0.5346  38: p = 0.9255  40: p = 0.4442  42: p = 0.0330  44: p = 0.7152  46: p = 0.5343  48: p = 0.8486 | Grooming behavior in 41-42th hour of observation:  Ctrl < VPA |
|  | C |  |  | Total distance moved (m) (light-off period) | Ctrl: 211.1 $\pm$ 22.50  VPA: 152.7 $\pm$ 11.41 | Mann-Whitney test  Ctrl vs VPA: p = 0.0921 | Light-off period long-term activity:  Ctrl = VPA |
|  | D |  |  | Distance moved (m) | Ctrl  2: 15.38 $\pm$ 3.09  4: 21.16 $\pm$ 4.35  6: 17.36 $\pm$ 2.78  8: 21.26 $\pm$ 3.70  10: 16.44 $\pm$ 2.98  12: 13.54 $\pm$ 2.33  14: 9.38 $\pm$ 1.92  16: 7.82 $\pm$ 1.92  18: 4.32 $\pm$ 1.34  20: 5.52 $\pm$ 1.58  22: 7.60 $\pm$ 1.16  24: 8.96 $\pm$ 1.45  26: 19.57 $\pm$ 3.04  28: 19.12 $\pm$ 2.41  30: 23.06 $\pm$ 2.95  32: 16.24 $\pm$ 2.23  34: 13.75 $\pm$ 2.15  36: 14.22 $\pm$ 2.59  38: 4.07 $\pm$ 1.09  40: 6.94 $\pm$ 1.55  42: 3.85 $\pm$ 0.95  44: 6.10 $\pm$ 1.44  46: 5.12 $\pm$ 1.39  48: 5.34 $\pm$ 1.13  VPA  2: 10.02 $\pm$ 1.73  4: 11.20 $\pm$ 2.37  6: 13.28 $\pm$ 2.18  8: 15.45 $\pm$ 2.43  10: 13.11 $\pm$ 2.76  12: 12.58 $\pm$ 1.45  14: 6.12 $\pm$ 1.48  16: 4.77 $\pm$ 1.19  18: 6.58 $\pm$ 2.12  20: 4.89 $\pm$ 1.32  22: 8.34 $\pm$ 2.54  24: 6.85 $\pm$ 2.21  26: 16.04 $\pm$ 2.78  28: 12.95 $\pm$ 1.69  30: 14.27 $\pm$ 1.58  32: 10.19 $\pm$ 1.84  34: 12.51 $\pm$ 2.24  36: 11.13 $\pm$ 1.51  38: 4.38 $\pm$ 1.19  40: 2.63 $\pm$ 1.04  42: 4.46 $\pm$ 1.37  44: 4.00 $\pm$ 0.93  46: 4.28 $\pm$ 1.20  48: 3.77 $\pm$ 0.75 | Multiple t-test  Ctrl vs VPA  2: p = 0.1677  4: p = 0.0722  6: p = 0.2757  8: p = 0.2241  10: p = 0.4284  12: p = 0.7441  14: p = 0.2068  16: p = 0.1978  18: p = 0.3640  20: p = 0.7711  22: p = 0.7817  24: p = 0.4205  26: p = 0.4097  28: p = 0.0573  30: p = 0.0223  32: p = 0.0548  34: p = 0.6942  36: p = 0.3400  38: p = 0.8481  40: p = 0.0387  42: p = 0.7089  44: p = 0.2555  46: p = 0.6621  48: p = 0.2830 | Activity in 29-30^th^ and 39-40^th^ hour of observation:  Ctrl > VPA |
|  | E |  |  | Total rearing time (min) (light-off period) | Ctrl: 34.74 $\pm$ 3.941  VPA: 28.84 $\pm$ 1.642 | Unpaired t-test  Ctrl vs VPA: p = 0.2122 | Light-off period long-term rearing:  Ctrl = VPA |
|  | F |  |  | Rearing time (s) | Ctrl  2: 145.62 $\pm$ 32.20  4: 216.35 $\pm$ 58.79  6: 138.59 $\pm$ 27.44  8: 208.13 $\pm$ 39.29  10: 174.66 $\pm$ 29.66  12: 162.34 $\pm$ 32.99  14: 112.42 $\pm$ 27.11  16: 89.36 $\pm$ 18.78  18: 68.51 $\pm$ 20.28  20: 101.57 $\pm$ 30.22  22: 93.14 $\pm$ 17.26  24: 106.00 $\pm$ 18.47  26: 202.58 $\pm$ 43.99  28: 183.12 $\pm$ 30.06  30: 201.99 $\pm$ 31.47  32: 161.44 $\pm$ 30.96  34: 150.39 $\pm$ 29.71  36: 139.47 $\pm$ 33.19  38: 37.97 $\pm$ 12.90  40: 69.95 $\pm$ 18.94  42: 51.44 $\pm$ 17.00  44: 85.96 $\pm$ 28.65  46: 88.19 $\pm$ 29.66  48: 67.15 $\pm$ 21.33  VPA  2: 116.52 $\pm$ 30.71  4: 109.51 $\pm$ 23.57  6: 121.44 $\pm$ 24.58  8: 171.98 $\pm$ 21.72  10: 197.42 $\pm$ 28.61  12: 122.86 $\pm$ 18.28  14: 49.32 $\pm$ 12.99  16: 96.30 $\pm$ 32.88  18: 132.50 $\pm$ 62.47  20: 79.86 $\pm$ 28.78  22: 59.78 $\pm$ 15.31  24: 55.42 $\pm$ 14.28  26: 131.68 $\pm$ 20.29  28: 195.77 $\pm$ 44.43  30: 156.04 $\pm$ 25.45  32: 99.56 $\pm$ 15.39  34: 150.27 $\pm$ 40.87  36: 157.08 $\pm$ 42.17  38: 58.38 $\pm$ 24.63  40: 34.42 $\pm$ 11.97  42: 64.39 $\pm$ 15.01  44: 35.14 $\pm$ 7.44  46: 47.99 $\pm$ 9.61  48: 47.37 $\pm$ 10.05 | Multiple t-test  Ctrl vs VPA  2: p = 0.5261  4: p = 0.1325  6: p = 0.6529  8: p = 0.4556  10: p = 0.5912  12: p = 0.3343  14: p = 0.0632  16: p = 0.8502  18: p = 0.3064  20: p = 0.6133  22: p = 0.1716  24: p = 0.0488  26: p = 0.1861  28: p = 0.8109  30: p = 0.2828  32: p = 0.1084  34: p = 0.9982  36: p = 0.7428  38: p = 0.4502  40: p = 0.1462  42: p = 0.5820  44: p = 0.1305  46: p = 0.2478  48: p = 0.4414 | Activity in 23-24^th^ hour of observation:  Ctrl > VPA |
| Sup. 1 | | Marble burying | n = 8 for Ctrl  n = 8 for VPA  2 month | Buried marbles | Ctrl: 11.13 $\pm$ 1.959  VPA: 13.13 $\pm$ 2.048 | Unpaired t-test  Ctrl vs VPA: p = 0.4920 | Digging:  Ctrl = VPA |
